# *MeABL5*, an ABA Insensitive 5-Like Basic Leucine Zipper Transcription Factor, Positively Regulates *MeCWINV3* in Cassava (*Manihot esculenta Crantz*)

**DOI:** 10.3389/fpls.2019.00772

**Published:** 2019-06-28

**Authors:** Jiao Liu, Xia Chen, Shuo Wang, Yuanyuan Wang, Yujun Ouyang, Yuan Yao, Ruimei Li, Shaoping Fu, Xinwen Hu, Jianchun Guo

**Affiliations:** ^1^Key Laboratory of Biology and Genetic Resources of Tropical Crops, Ministry of Agriculture and Rural Affairs, Institute of Tropical Bioscience and Biotechnology, Chinese Academy of Tropical Agricultural Sciences, Haikou, China; ^2^Institute of Tropical Agriculture and Forestry, Hainan University, Haikou, China; ^3^Dazhou Mingrenyuan Middle School, Dazhou, China

**Keywords:** cassava, basic leucine zipper transcription factor, abscisic acid insensitive 5, cell wall invertase, promoter, abiotic stress

## Abstract

The basic leucine zipper (bZIP) transcription factor family plays crucial roles in multiple biological processes, especially stress responses. Cassava (*Manihot esculenta* Crantz) is an important tropical crop with a strong tolerance to environmental stresses such as drought, heat, and low-fertility environments. Currently, limited information is available regarding the functional identification of bZIP transcription factors in response to abiotic stress in cassava. Herein, a gene encoding an ABA Insensitive 5 (ABI5)-like transcription factor, designated as *MeABL5*, was identified in cassava. Sequence and phylogenetic analysis showed that MeABL5 is a cassava bZIP transcription factor that is not included in the previously identified cassava bZIP family members, belongs to subfamily A, and has high sequence similarity to ABI5-like proteins. Subcellular localization and transactivation assays revealed that MeABL5 was a nuclear-localized protein and possessed transactivation activity. Furthermore, MeABL5 was able to specifically interact with the ABRE cis-element in the promoter of the cassava major cell wall invertase gene, *MeCWINV3, in vitro* and *in vivo. MeABL5* and *MeCWINV3* exhibited similar expression patterns in various organs or tissues and under abiotic stress in cassava. The expressions of *MeABL5* and *MeCWINV3* within cassava plantlets were both induced by exogenous abscisic acid (ABA), gibberellic acid (GA3), methyl jasmonate (MeJA), and heat. Overexpression of *MeABL5* increased the activity of the *MeCWINV3* gene, and the up-regulated expressions of *MeCWINV3* were significantly activated under ABA-, salicylic acid (SA)-, and MeJA-induced conditions. Overall, these results suggest that *MeABL5* is a positive regulator of *MeCWINV3* and might participate in the robust resistance of cassava in response to abiotic stress. This study also provides a foundation for further research on ABA-mediated and stress-related signaling pathways in cassava.

## Introduction

Cassava (*Manihot esculenta* Crantz), the third most important crop after rice and maize in Asia, Africa, and Latin America, provides nourishment for 800 million people across tropical and subtropical regions of the world (Lebot et al., 2009; Oliveira et al., 2014). Cassava has a tolerance for environmental stresses such as drought, heat, and low-fertility soil due to its efficient use of heat, light, and water resources (Maran et al., 2014; Zeng et al., 2014).

Cell wall invertases (CWIs) are glycosylated enzymes, irreversibly convert sucrose into hexoses, and generate a Suc gradient that is essential for phloem unloading and carbon partitioning. CWIs play principal roles in the development of plant tissues such as roots, flowers, fruits, and seeds, as well as in responses to adverse stresses *via* sugar metabolism and signaling pathways in plants (Doidy et al., 2012; Ruan, 2014). Numerous previous studies have revealed that the transcripts of CWIs are significantly up-regulated in source tissues upon pathogen-, drought (or water-deficit)-, heat-, and abscisic acid (ABA)-induced stresses (Rivero et al., 2007; Zhao et al., 2015; Clauw et al., 2016). For instance, CWIs promote tomato (*Solanum lycopersicum*) fruit survival under heat stress by suppressing ROS-independent cell death to enhance sucrose import and catabolism, *HSP* expression, and auxin response and biosynthesis (Liu et al., 2016). The *CsINV* genes from the tea plant participate in regulating plant growth and development and regulate the carbohydrate allocation and the ratio of hexose and sucrose to improve the resistance of the leaves and roots to various abiotic stresses (Qian et al., 2016). The plant hormone ABA enhances sugar accumulation in crop sink organs and Pan et al. (2010) have reported that ABA activates acid invertases in the developing grape berry. In cassava, genome-wide identification, expression, and activity analysis of the *CWI* family genes were reported in our previous study (Yao et al., 2014). There are six CWIs in cassava; the activity of MeCWINV1 and MeCWINV3 was higher than that of other MeCWINVs in the apoplastic space of cassava source and sink organs (Yao et al., 2014). *MeCWINV3* encodes a typical CWI in cassava. It is highly expressed in leaves, especially mature leaves, in response to diurnal rhythm, and affects cassava productivity *via* regulating sugar allocation from source to sink (Yan et al., 2019). At present, researches of the function and regulatory mechanism about *MeCWINV3* have been almost focused on the hydrolysis of sugar during phloem unloading to control plant development and sink strength, but have rarely been studied in response to various stresses in cassava.

The basic leucine zipper (bZIP) transcription factor family is one of the largest and most conserved families in plants and takes part in multiple biological processes, such as organ differentiation, seed germination, flowering induction, and vascular development (Jakoby et al., 2002). These proteins are also involved in light, sugar, and hormone signaling pathways and play crucial roles in the regulation of the abiotic and biotic stress responses (Jakoby et al., 2002; Lindemose et al., 2013). Plant bZIP transcription factors contain a basic region that binds DNA and a leucine zipper dimerization motif to create an amphipathic helix and regulate multiple processes, especially stress responses (Jakoby et al., 2002; Wei et al., 2012; Liu et al., 2014). In many plants, such as Arabidopsis (*Arabidopsis thaliana*) (Jakoby et al., 2002), grapevine (*Vitis vinifera*) (Liu et al., 2014), cucumber (*Cucumis sativus*) (Baloglu et al., 2014), maize (*Zea mays*) (Wei et al., 2012), and cassava (*M. esculenta* Crantz) (Hu et al., 2016b), bZIP members can be divided into 10 groups (named A, B, C, D, E, F, G, H, I, and S) according to the sequence similarity within the basic region. Studies have revealed that most of the members of group A can bind different ABA-responsive element (ABRE)-containing promoters and are involved in ABA or stress signaling (Finkelstein and Lynch, 2000; Kang et al., 2002; Hossain et al., 2010; Li et al., 2016b). In cassava, a total of 77 bZIP proteins are known, named MebZIP1 to MebZIP77, 15 of which (MebZIP5, 11, 14, 15, 27, 34, 35, 48, 49, 61, 65, 66, 67, 70, and 71) belong to group A (Hu et al., 2016b). Additionally, ABA Insensitive 5 (ABI5) is a member of the bZIP transcription factor subfamily A. It shares putative phosphorylation sites at its N-terminus, a conserved bZIP domain, and a putative sumoylation site at its C-terminus (Kim et al., 2002; Zhou et al., 2013). ABI5 functions in the core ABA signaling pathway, which is composed of RCAR/PYR/PYL ABA receptors, PP2C phosphatases, and SnRK2 kinases (Choi et al., 2000; Uno et al., 2000). It has been reported that ABI5 is involved in ABA-mediated or stress signaling responses and regulates the expression of stress-responsive genes that contain the ABRE motif within their promoter region (Finkelstein and Lynch, 2000; Zou et al., 2008; Hossain et al., 2010; Zhou et al., 2013). Similarly, OsABI5 binds to ABRE (G-box), is involved in ABA signal transduction, and regulates fertility and stress responses, possibly acting as an ABA-dependent negative regulator of stress tolerance in rice (*Oryza sativa*) (Zou et al., 2008). In cabbage (*Brassica oleracea*), BolABI5 activates expression of target genes through binding to ABRE elements in their promoters, indicating its role in abiotic stress adaptation (Zhou et al., 2013).

In the present study, we describe the isolation and characterization of an ABI5-like bZIP transcription factor, *MeABL5*, from the cassava (*M. esculenta* Crantz) cultivar SC8 through a yeast one-hybrid (Y1H) screen using the promoter of *MeCWINV3* as a bait. The sequence structure, evolutionary relationships, subcellular fate, and transactivation activity of the MeABL5 protein were studied. Moreover, the interaction of MeABL5 and the *MeCWINV3* promoter was confirmed *in vitro* and *in vivo*. The expression profiles of *MeABL5* and *MeCWINV3* in cassava plant organs or tissues and in response to abiotic stresses were examined using qRT-PCR. To investigate the transcription regulation between *MeABL5* and *MeCWINV3* under abiotic stresses, overexpression of *MeABL5* in cassava friable embryogenic calli (FECs) was analyzed. These results are the basis for further studies on *MeABL5* and its roles in the cassava response to abiotic stresses.

## Materials and Methods

### Plant Materials

Cassava (*M. esculenta* Crantz, cultivar SC8) was planted in the experimental plantation of the Chinese Academy of Tropical Agriculture Sciences (Wenchang, Hainan, China) under natural conditions. The leaves, stems, and fibrous roots were collected at 90 days after planting (DAP); the flowers, storage roots, storage root phloems, and xylems were collected at 200 DAP; and the fruits were collected at 220 DAP (20 days after flowering). For exogenous hormone and abiotic stress treatments, 45-day-old tissue culture plantlets of cassava were set under the culture conditions of 25°C, for a 16-h light/8-h dark photoperiod; after the 8-h dark period, plantlets were treated with hormones (100 μM ABA, 2 mM SA, 100 μM GA3, or 100 μM MeJA) and abiotic stresses (20% PEG2000 artificial drought, 4°C low temperature or 42°C high temperature), followed by sampling at 1, 3, 6, 12, and 24 h. The parallel mock (treated with H_2_O) in the different sampling times were used to normalize the expression. The untreated plantlets at 0 h were collected as control. All materials were immediately frozen in liquid nitrogen and stored at −80°C.

Tobacco (*Nicotiana benthamiana*) seedlings were grown in controlled environment growth chambers under conditions of 16-h light (26°C)/8-h dark (21°C) cycles and 70–80% relative humidity.

### Y1H Assays

Y1H assays were performed using the Matchmaker Gold Y1H System (Clontech, CA, USA) according to the manufacturer’s protocol. Total RNAs were extracted using Trizol (Invitrogen) from the mixed leaves, stems, flowers, fruits, and storage roots of cassava. Synthesis of the cassava double-stranded cDNA library was constructed according to the instructions of the cDNA Synthesis Kit (Fermentas). The 1,160-bp *MeCWINV3* promoter (GenBank Accession No. KC905170) and the different fragments P1 (−1 to −400), P2 (−401 to −800), P2 without the ABRE *cis*-element, and P3 (−801 to −1,160) of *MeCWINV3* promoter were isolated from cassava genomic DNA with appropriate primers (Supplementary Table S1) and inserted into the pAbAi vector (Clontech) at the *Sal* I/*Kpn* I sites to construct the bait vector p*MeCWINV3*-AbAi. Then, the p*MeCWINV3*-AbAi bait vector was linearized with BstBI and transformed into the yeast Y1HGold strain to generate the Bait-Reporter yeast strain, Y1HGold[p*MeCWINV3*-AbAi]. The cassava cDNAs and the *Sma* I-linearized prey vector, pGADT7-Rec, were introduced into the Y1HGold[p*MeCWINV3*-AbAi] yeast strain and then cultivated on SD/−Leu medium with 100 ng/ml Aureobasidin A (AbA) at 30°C for 3 days. Positive colonies were identified by colony PCR and sequence analysis.

To confirm the binding specificity of MeABL5 with the *MeCWINV3* promoter, the MeABL5 coding sequence (CDS) and the MeABL5 CDS region excluding the bZIP domain (MeABL5ΔbZIP) were amplified from the leaf cDNA of cassava cultivar SC8 (primers shown in Supplementary Table S2) and then were ligated into pGADT7 vector with *Nde* I and *Bam*H I and named as pGADT7-MeABL5 or pGADT7-MeABL5ΔbZIP. Both the p*MeCWINV3*-AbAi bait vectors and pGADT7-MeABL5 prey vectors were cotransformed into the Y1HGold yeast strain. pGADT7 + p*MeCWINV3*-AbAi, pGADT7-*MeABL5* + pAbAi, and pGAD-*Rec2-53* + p*53HIS2*-AbAi were used as controls. The transformed cells were grown on SD/−Leu selective medium containing 0 or 150 ng/ml AbA at 30°C for 3 days.

### Sequence Alignment and Phylogenetic Analysis

The deduced amino acid sequences of MeABL5 in *M. esculenta* and its orthologues, AtABI5 (*A. thaliana*, AAD21438.1), BolABI5 (*B. oleracea*, JX870620.1), OsABI5 (*O. sativa*, ABM90395.1), and ZmABI5 (*Z. mays*, NP_001150949), were analyzed by DNAman 6.0 software (Lynnon Biosoft, Quebec, QC, Canada).

A total of 75 Arabidopsis bZIP sequences and 77 cassava bZIP sequences were obtained from the UniProt^1^ and Phytozome^2^ databases, respectively. The phylogenetic tree was constructed by employing MEGA using the neighbor-joining method with 1,000 bootstrap replicates (Kumar et al., 2016).

### Real-Time Quantitative Polymerase Chain Reaction

Total RNAs were extracted from various cassava organs or tissues, abiotic stress- and exogenous hormone-treated plantlets, and wild-type and transgenic cassava FECs using Trizol reagents (Invitrogen). Complementary DNAs were synthesized from total RNA using the reverse transcriptase kit (Takara, China). The relative mRNA expressions of *MeABL5* and *MeCWINV3* were analyzed by real-time quantitative PCR (qRT-PCR). Primer sequences are listed in Supplementary Tables S1 and S2. qRT-PCR was conducted using the SYBR Premix Taq Kit (TaKaRa, Dalian, China) and the reactions were performed as described previously (Geng et al., 2017). The cassava tubulin gene (Phytozome name: 4.1_007598m.g) mRNA was amplified as an internal control with the primer pairs 5′-ATGCGGTTCTTGATGTTGTTC-3′ and 5′-TCGGTGAAGGGAATACAGAGA-3′ to calculate the relative expression using the 2^−ΔΔCt^ method (Livak and Schmittgen, 2001). Three technical replicates were analyzed for each biological sample.

### Subcellular Localization Analysis

The CDS of *MeABL5* without the termination codon was amplified with *Sal* I and *Bam*H I restriction sites, and then cloned into the modified plant expression vector pCAMBIA1300-GFP to generate the construct CaMV35S::MeABL5-GFP. The CaMV35S::MeABL5-GFP construct and control (empty vector pCAMBIA1300-GFP) were, respectively, introduced into the *Agrobacterium tumefaciens* strain GV3101 by freeze–thaw method. The Agrobacterium cells harboring different constructs were cultured in YEP medium containing 50 mg/L kanamycin and 50 mg/L rifampicin and grown overnight at 28°C. Bacteria were collected by centrifugation (6,000 rpm) and resuspended to an OD_600_ of 1.0 in infiltration medium [10 mM MgCl_2_, 10 mM MES (pH 5.6), and 200 μM acetosyringone] at room temperature for 2 h before injection into the leaves of 4- to 5-week-old tobacco (*N. benthamiana*) plants. The agroinfltrated tobacco plants were maintained under normal growth conditions for 72 h. For microscopic analyses, the infiltrated leaf discs were cut and visualized the GFP-fluorescence signals using an Olympus FluoView™ FV1000 confocal microscope with an Argon laser (Olympus, Tokyo, Japan). GFP was excited at 488 nm, and the emitted light was captured at 520 nm. Chlorophyll autofluorescence was excited at 559 nm, and the emitted light was captured at 618 nm. Images were captured digitally and processed using the Olympus FluoView™ Application (FV10-ASW version: 2.0\u00B0C). Three independent experiments were performed, six plants were used for each agrobacterium-injected treatment, and 40–50 cells were examined for each experiment.

### Transcriptional Activation Analysis

For transactivation assays, the complete CDS of *MeABL5* was cloned into the pGBKT7 vector (Clontech, CA, USA) to construct the pGBKT7*-MeABL5* vector. pGBKT7*-MeABL5*, pGBKT7-*p53* + pGADT7-*largeT* (positive control), and pGBKT7 (negative control) were introduced into the yeast strain AH109, respectively. The transformed cells were separately spotted onto SD/−Trp + 10 mg/ml X-α-gal medium, which were then incubated for 36 h at 30°C in the dark. The transactivation activity was evaluated according to the color of the resulting colonies.

### Protein Expression and Purification

The open reading frames (ORFs) of *MeABL5* were amplified with added *Hind* III and *Xho* I restriction sites, and then fused to the maltose-binding protein (MBP) in the pET28a-MBP vector (GenScript, Nanjing, China), which generated the pET28a-MBP-MeABL5. The pET28a-MBP vector contains an MBP that can promote the folding of target proteins and increase their expression level and solubility in prokaryotic expression systems. The MBP-tagged MeABL5 protein was expressed in *Escherichia coli* BL21 (DE3) by inducing with 1.0 mM isopropyl-β-D-thiogalactopyranoside (IPTG) at 37°C for 4 h, followed by purification using a Ni column (GenScript, Nanjing, China) according to the manufacturers’ instructions. The MBP-tagged protein was then cleaved with TEV Protease (GenScript, Nanjing, China) to remove the MBP affinity tag from the fusion protein. The recombinant MeABL5 protein was dialyzed and sterilized by a 0.22-μm filter before being stored in aliquots. The concentration was determined by Bradford protein assay with bovine serum albumin (BSA) as a standard. The protein purity and molecular weight were determined by standard SDS-PAGE along with Western blot.

### Electrophoretic Mobility Shift Assay

An electrophoretic mobility shift assay (EMSA) was performed using the EMSA kit (Invitrogen, USA). The 1,160-bp double-stranded *MeCWINV3* promoter DNA fragment (GenBank Accession No. KC905170) was isolated from cassava genomic DNA with the primers (listed in Supplementary Table S1). The 60-bp p*MeCWINV3*-WT sequence containing the ABRE *c*is-element and the 54-bp p*MeCWINV3*-MT sequence without the ABRE *c*is-element were synthesized by Sangon Biotech (Sangon, China) and then annealed to double-stranded oligonucleotides at 95°C for 6 min, respectively. The DNA-protein binding reactions were performed by incubating the purified recombinant MeABL5 protein (0, 100, 150, 200, or 250 ng) with 500 ng of the *MeCWINV3* promoter DNA fragment or the double-stranded WT/MT oligonucleotides at room temperature for 30 min (250 ng BSA was used as the negative control), followed by analysis *via* polyacrylamide gel electrophoresis. The gel was stained with SYBR Green EMSA stain for visualizing DNA, and the same gel was stained with SYPRO Ruby EMSA stain for monitoring proteins as previously described (Guo et al., 2018).

### Dual-Luciferase Assay

The dual-luciferase assay was performed according to Hellens’s method (Hellens et al., 2005). The full-length *MeABL5* ORF was amplified from cassava cDNA and cloned into the effector vector pGreen II 62-SK, generating pGreen II 62-SK-*MeABL5*. The *MeCWINV3* promoter fragment was amplified with specific primers (Supplementary Table S1) and cloned into the pGreen II 0800-LUC reporter vector using *Kpn* I/*Bam*H I to generate pGreenII-*MeCWINV3*-LUC. The two vectors were separately cotransformed into *A. tumefaciens* GV3101 cells with the pSoup vector. The transformed GV3101 cells with a 2:1 effector/reporter volume ratio were used for infiltration into young tobacco leaves as previously described (Hu et al., 2016a). After culturing for 3 days, total protein was extracted from the infected area. Firefly luciferase and the reference Renilla luciferase were detected using the Dual-Luciferase Reporter Assay System (Promega) following the manufacturer’s manual. Three biological repeats were measured. Data were analyzed by one-way ANOVA. Differences were accepted as significant at *p* < 0.01.

### Transformation and Treatment of Cassava Friable Embryogenic Callus

*A. tumefaciens* strain GV3101 carrying the *MeABL5* overexpressing vector CaMV35S::MeABL5 was used for cassava SC8 FEC transformation. The GV3101 strain harboring CaMV35S::MeABL5 was cultured in YEP medium containing 50 mg/L kanamycin and 50 mg/L rifampicin and grown overnight at 28°C to reach an OD_600_ of 0.75**–**1.0. Bacteria were spun down and resuspended in liquid GD medium supplemented with 200 μM acetosyringone (Sigma, USA) to an OD_600_ of 0.25. Three-month-old FECs (100 mg) were co-cultivated with the suspension for 30 min, transferred on sterilized paper towel for 5 min to remove excess bacteria, and co-cultivated on GD medium for 3 days in the dark at 22°C. Then, agro-inoculated FECs were washed three times with liquid GD medium containing 500 mg/L carbenicillin, transferred to the GD media supplemented with 250 mg/L carbenicillin and 5 mg/L hygromycin, and incubated at 28°C at a 16/8-h photoperiod for 7 days. For exogenous hormone treatments and abiotic stresses, the untransformed wild type and agro-inoculated FECs were cultured in liquid GD medium and then treated with hormones (100 μM ABA, 2 mM SA, 100 μM GA3, or 100 μM MeJA) and abiotic stresses (20% PEG2000, 4 or 42°C) for 12 h under gentle shaking (40 rpm) and harvested by centrifugation. Three independent transformation experiments were performed. The relative mRNA expressions of *MeABL5* and *MeCWINV3* were analyzed by qRT-PCR as described above.

## Results

### Isolation and Molecular Characterization of *MeABL5* in Cassava

**Figure 1:**
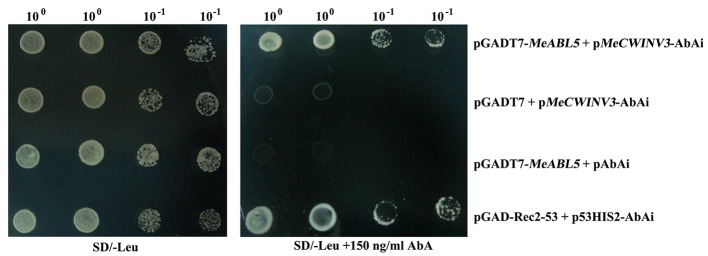
Activation of the MeCWINV3 promoter in yeast by MeABL5. pGADT7-MeABL5 + pMeCWINV3-AbAi shows that MeABL5 can bind to promoter of MeCWINV3; pGADT7-Rec2-53 + p53HIS2-AbAi is the positive control and pGADT7 + pMeCWINV3-AbAi and pGADT7-MeABL5 + pAbAi are the negative controls. Yeast cells were grown in SD/−Leu selective medium containing 0 or 150 ng/ml Aureobasidin A (AbA) for 3 days at 30°C.

**Figure 2:**
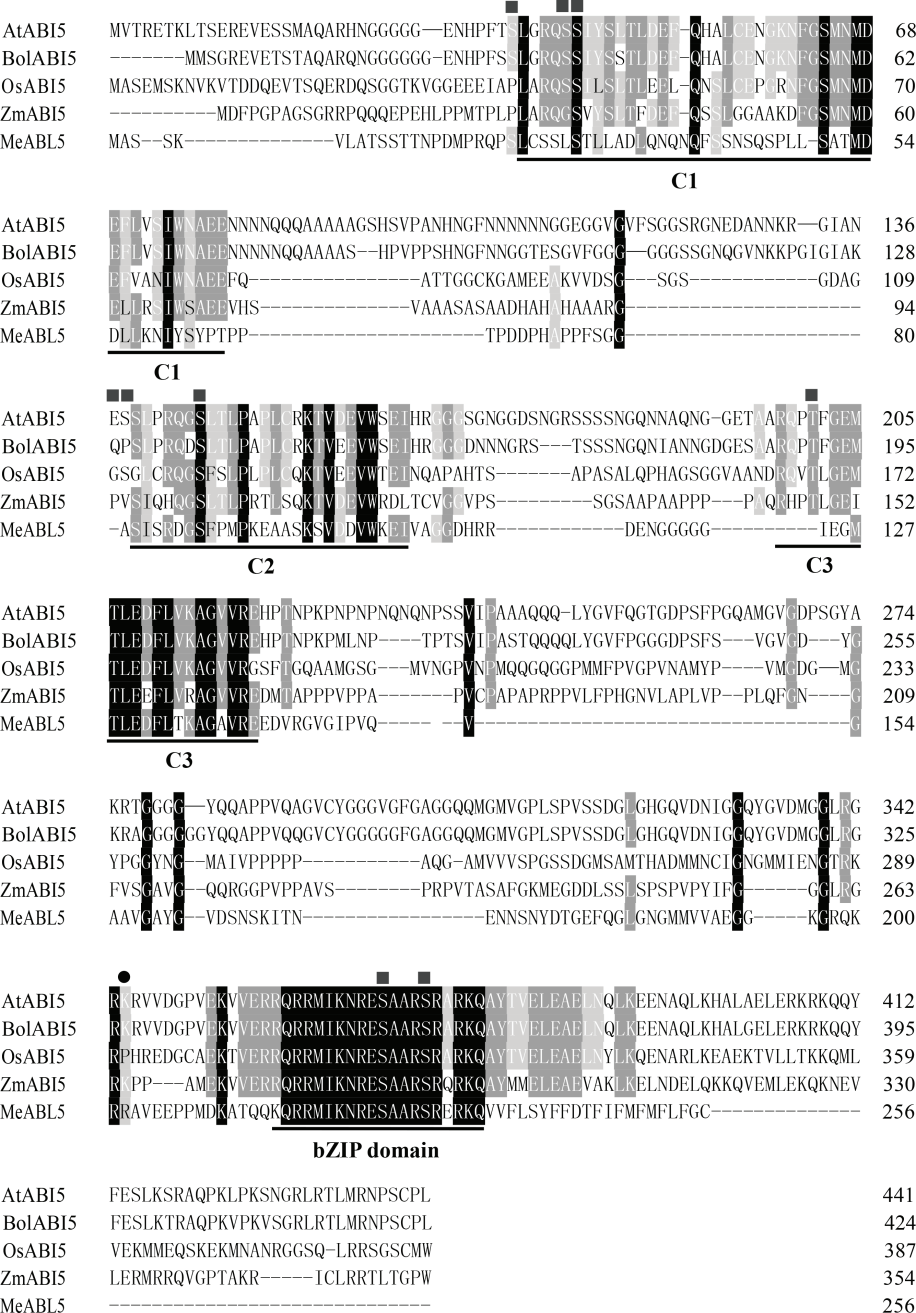
Alignment of MeABL5 and other ABI5 proteins. The amino acid sequence of MeABL5 was aligned with AtABI5 (Arabidopsis thaliana, AAD21438.1), BolABI5 (*Brassica oleracea*, JX870620.1), OsABI5 (*Oryza sativa*, ABM90395.1), and ZmABI5 (*Zea mays*, NP_001150949). The squares and circles mark phosphorylation and ubiquitination sites, respectively. C1, C2, and C3, conserved regions; bZIP, basic leucine zipper domain.

**Figure 3:**
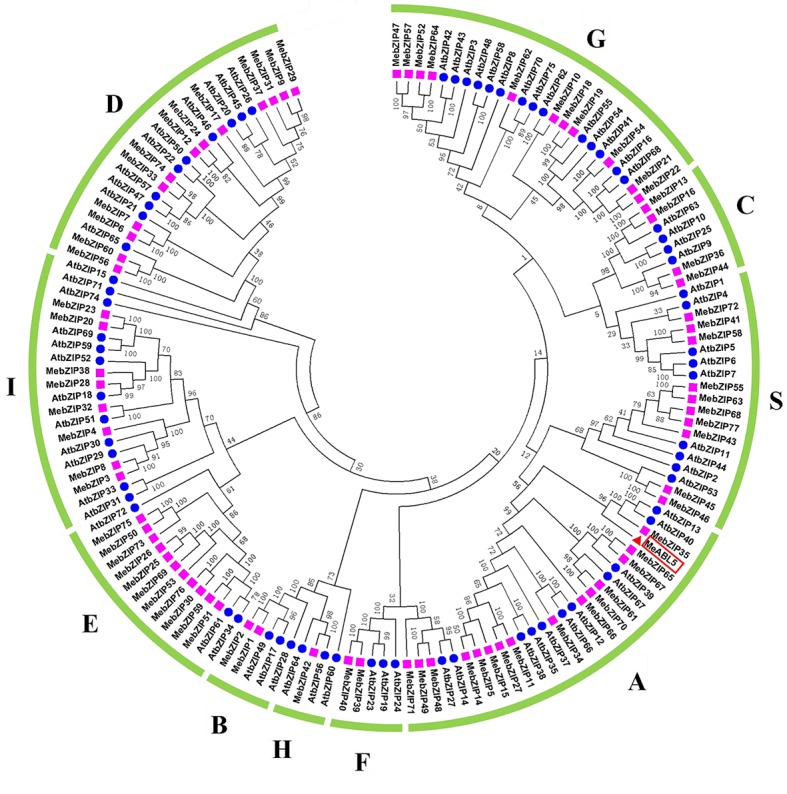
Phylogenetic analysis of MeABL5 with bZIP proteins from cassava and Arabidopsis. A total of 77 bZIPs from cassava, 75 bZIPs from Arabidopsis, and MeABL5 were used to create the NJ tree with 1,000 bootstraps using MEGA software from CLUSTAL W alignments. The bZIP proteins are grouped into 10 subfamilies (A, B, C, D, E, F, G, H, I, and S), and MeABL5 was classified into subfamily A.

To understand the transcriptional regulatory mechanism of a key CWI gene, *MeCWINV3*, in cassava, a Y1H assay using a cDNA library normalized from leaves, stems, flowers, fruits, and storage roots of cassava was used to screen for candidate transcription factors that could bind to the *MeCWINV3* promoter. A total of 21 positive colonies were obtained, sequenced, and BLAST-searched in GenBank. One cDNA, designated as *MeABL5* (GenBank Accession No. MK122661, https://www.ncbi.nlm.nih.gov/nuccore/MK122661), that could bind to the *MeCWINV3* promoter as determined by a Y1H one-to-one interaction analysis (Figure 1) was identified and isolated from cassava. *MeABL5* has 99.6% identity with a predicted *M. esculenta* ABSCISIC ACID-INSENSITIVE 5-like protein (GenBank Accession No. XM_021754330). Sequence analysis showed that *MeABL5* has a 771-bp ORF encoding a deduced protein of 256 amino acid residues with a predicted molecular mass of 27.67 kDa (Supplementary Figure S1). The deduced MeABL5 protein contains the conserved regions of ABI5 and ABI5-like proteins, such as the three conserved regions, C1, C2, and C3, that include phosphorylation sites at the N-terminus, a conserved bZIP domain, and a putative ubiquitination site at the C-terminus (Figure 2; Supplementary Figure S1). A total of 77 bZIP transcription factor genes were previously identified in cassava (Hu et al., 2016b), but the *MeABL5* gene was not included among these. A phylogenetic analysis using the full-length amino acid sequences of 75 bZIP proteins from Arabidopsis (Jakoby et al., 2002) and 77 from cassava (Hu et al., 2016b) indicated that *MeABL5* is clustered into the bZIP subfamily A and shows the closest homology to AtbZIP13 (AT5G44080) and AtbZIP40/GBF4 (AT1G03970), which were previously classified as the subfamily A bZIPs (Figure 3).

### MeABL5 Is a Nuclear-Localized Protein

**Figure 4:**
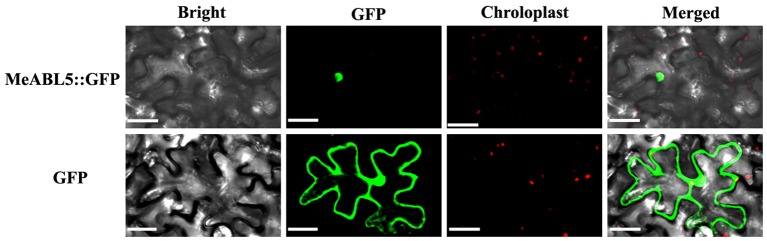
Nuclear localization of the MeABL5 protein. Green fluorescent protein (GFP) fluorescence is shown in green and chlorophyll autofluorescence is shown in red as a chloroplast marker. Top row/bottom row: the corresponding bright field, GFP fluorescence, chlorophyll autofluorescence, and merged fluorescent images of MeABL5-GFP/GFP control. Bar = 30 μm.

MeABL5 was predicted to be localized in the nucleus based on predictions by the TargetP software. To examine the subcellular localization of MeABL5, the CDS of *MeABL5* was cloned into the pCAMBIA1300-GFP vector, and the resulting construct was named CaMV35S::MeABL5-GFP. Confocal images indicated that the MeABL5-GFP fusion proteins were exclusively distributed in the nucleus of tobacco mesophyll cells, whereas GFP was observed throughout the entire cell with the control vector without MeABL5 (Figure 4). These results demonstrate that MeABL5 is a nuclear-localized protein.

### MeABL5 Exhibits Transactivation Activity

**Figure 5:**
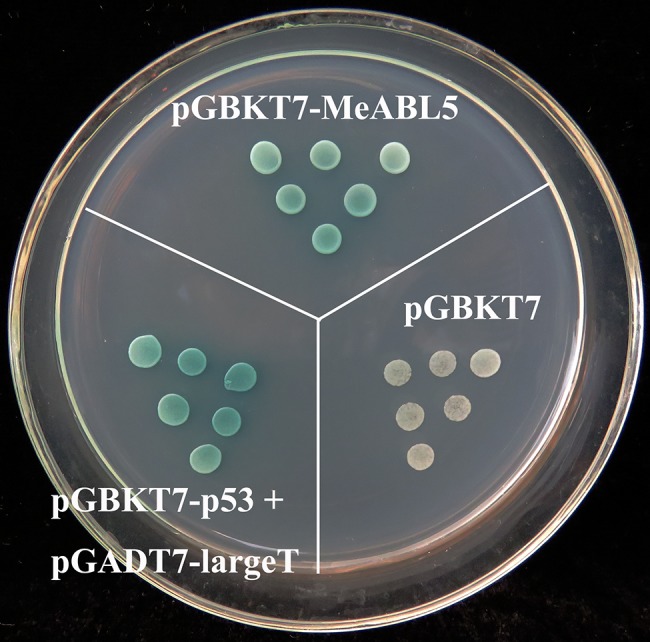
The transactivation activity of the MeABL5 protein in yeast. MeABL5 was inserted into the pGBKT7 vector and transformed into the AH109 yeast strain. The empty vector, pGBKT7, and pGBKT7-p53 + pGADT7-largeT were also transformed into AH109 as a negative and positive control, respectively. The yeast cells were spotted on plates with SD/−Trp selective medium containing 10 mg/ml X-α-gal.

The bZIP transcription factors have been shown to possess transactivation activity. A yeast two-hybrid (Y2H) in the yeast strain AH109 was used to investigate the transactivation activity of MeABL5. The CDS of MeABL5 was cloned into the GAL4 DNA-BD vector, pGBKT7, which resulted in a pGBKT7-MeABL5 fused vector. The pGBKT7-MeABL5, pGBKT7 empty vector (negative control), and pGBKT7-p53 + pGADT7-largeT (positive control) were transformed into AH109 and then screened on SD/−Trp selective medium containing 10 mg/ml X-α-gal. X-α-gal is a chromogenic substrate for α-galactosidase that is used to indicate the transactivation ability of fused proteins. Secretion of α-galactosidase in response to GAL4 activation leads to the hydrolysis of X-α-gal in the medium, which causes the yeast colonies to develop a blue color (Aho et al., 1997). The yeast cells harboring the pGBKT7-MeABL5 construct or the positive control vector grew as blue colonies on the SD/−Trp medium containing X-α-gal, while the yeast with the empty vector, pGBKT7, grew only as white colonies on the same selection medium (Figure 5). This confirmed that MeABL5 exhibited transactivation activity in yeast. These data were therefore consistent with its predicted function as a transcription factor.

### MeABL5 Interacts With the *MeCWINV3* Promoter *in vitro*

**Figure 6:**
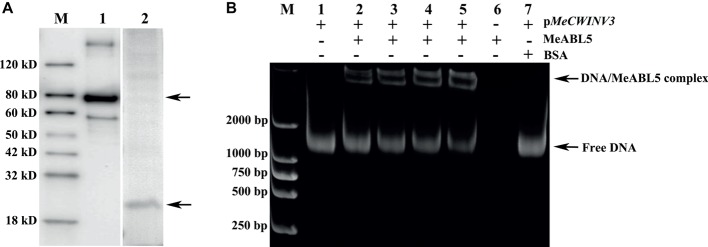
EMSA of the interaction between MeABL5 and the MeCWINV3 promoter. **(A)** Expression of the MeABL5 protein in E. coli BL21 (DE3). M: Protein marker; Lane 1: the MBP-tagged MeABL5 protein (top arrow). Lane 2: the target MeABL5 protein with the MBP affinity tag removed by TEV protease (bottom arrow). **(B)** Analysis of the MeABL5 interaction with the MeCWINV3 promoter by EMSA. The promoter of MeCWINV3 with the MeABL5 protein was stained with SYBR Green EMA for visualization of DNA. M: DNA marker. Lane 1: the promoter of MeCWINV3 DNA (500 ng) only; Lanes 2–5: the promoter of MeCWINV3 DNA (500 ng) with increasing amounts of MeABL5 protein (100, 150, 200, and 250 ng); Lane 6: 250 ng MeABL5 protein only; Lane 7: the promoter of MeCWINV3 DNA (500 ng) and 250 ng bovine serum albumin (BSA). The arrows indicate the MeABL5–DNA complex or free DNA.

To investigate whether MeABL5 interacts with the promoter of *MeCWINV3 in vitro*, an EMSA assay was performed using the recombinant MeABL5 protein and 1,160-bp *MeCWINV3* promoter nucleotide probes. The MeABL5 protein was purified from *E. coli* harboring the vector pET28a-MBP-MeABL5 (Figure 6A). The DNA–protein binding reaction was performed by incubating the purified recombinant MeABL5 protein (0, 100, 150, 200, or 250 ng) with 500 ng of the 1,160-bp double-stranded *MeCWINV3* promoter DNA fragment at room temperature for 30 min. As shown in Figure 6B, MeABL5 was able to bind to the *MeCWINV3* promoter and cause a shift in mobility. Moreover, with increasing concentrations of the MeABL5 protein, the quantity of the shifted protein–DNA complexes gradually increased, while the quantity of free DNA decreased (Figure 6B, lanes 1–5). The results showed that MeABL5 was able to recognize and interact with the *MeCWINV3* promoter *in vitro*.

### MeABL5 Binds to the ABRE *cis*-Element of the *MeCWINV3* Promoter

**Figure 7:**
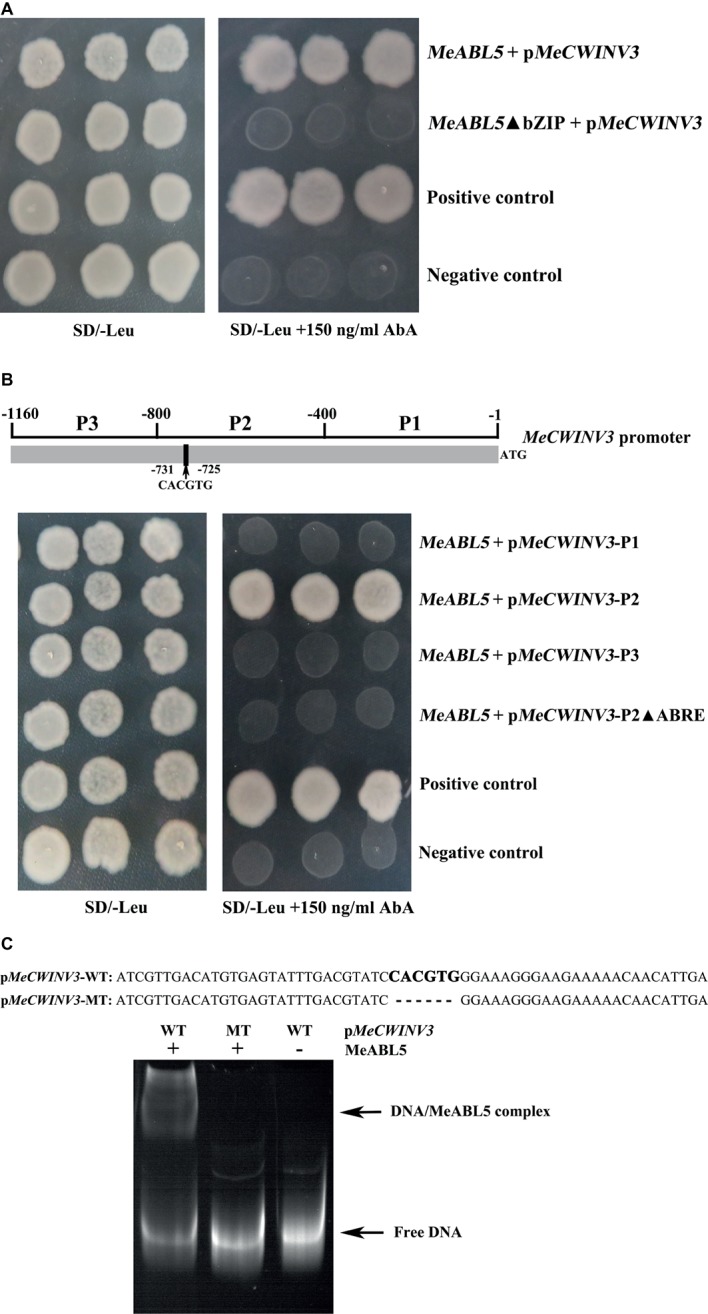
MeABL5 binds to the ABRE cis-element in the MeCWINV3 promoter. **(A)** Yeast-one hybrid (Y1H) assay of the core binding site in MeABL5 binds to the MeCWINV3 promoter. The full-length MeABL5 and the bZIP domain deletion (MeABL5ΔbZIP) sequences were inserted into the pGADT7 vector. Yeast cells carried pGADT7-MeABL5/MeABL5ΔbZIP + pMeCWINV3-AbAi, positive control (pGADT7-Rec2–53 + p53HIS2-AbAi), and negative control (pGADT7 + pMeCWINV3-AbAi), were grown in SD/−Leu selective medium containing 0 or 150 ng/ml AbA, for 3 days at 30°C. **(B)** Analysis of the MeABL5 interaction with the ABRE cis-element of MeCWINV3 promoter by Y1H assay. The different fragments P1 (−1 to −400), P2 (−401 to −800), P2 without the ABRE cis-element, and P3 (−801 to −1,160) of MeCWINV3 promoter were inserted into the pGBKT7 vector. Yeast cells carried pGADT7-MeABL5 + pMeCWINV3 (P1 to P3, P2ΔABRE)-AbAi, positive control (pGADT7-Rec2–53 + p53HIS2-AbAi), and negative control (pGADT7-MeABL5 + pAbAi) were grown in SD/−Leu selective medium containing 0 or 150 ng/ml AbA, for 3 days at 30°C. **(C)** Analysis of the MeABL5 interaction with the ABRE cis-element of MeCWINV3 promoter by EMSA. pMeCWINV3-WT: 60 bp DNA sequence contained the ABRE cis-element on the MeCWINV3 promoter; the bold CACGTG represents the ABRE cis-element binding site. pMeCWINV3-MT: 54 bp DNA sequence without the ABRE cis-element on the MeCWINV3 promoter. The + and − indicate the presence and absence of 250 ng MeABL5 protein, respectively. The arrows indicate the MeABL5–DNA complex or free DNA.

The basic region containing an N-X7-R/K-X9 motif in plant bZIP transcription factors is reported to directly bind to special DNA and possess the transactivation activity (Kim et al., 2002; Pradeep et al., 2016). To further verify the interaction of MeABL5 with the *MeCWINV3* promoter, the bZIP domain deletion (*MeABL5*ΔbZIP) sequence of *MeABL5* as well as its full-length CDS were inserted into the pGADT7 vector for use in a Y1H assay. Consistent with the previous observations on BolABI5 (Zhou et al., 2013), BrABI5a/b (Bai et al., 2016), and wheat bZIP2 (Pradeep et al., 2016), the Y1HGold yeast cells transformed with *MeABL5*ΔbZIP (deleted the conserved bZIP domain) showed less binding activity than those transformed with the full-length MeABL5 (Figure 7A). This result indicates that the conserved bZIP domain of MeABL5 is critical for its recognition and interaction with the *MeCWINV3* promoter.

Furthermore, to determine the exact binding region of the MeABL5 protein, a 1,160-bp *MeCWINV3* promoter sequence upstream of the translation initiation codon (ATG) was analyzed using the PLACE and PlantCARE bioinformatic tools. The promoter region (from −731 to −725 bp) of *MeCWINV3* contains a potential ABRE *cis*-element CACGTG (Figure 7B; Supplementary Figure S2). The *MeCWINV3* promoter was cleaved into three segments P1, P2, and P3 (each segment is approximately 400 bp in length, and P2 contains the ABRE *cis*-element). The fragments of P1, P2, and P3, and P2 without the ABRE *cis*-element were inserted into the pGBKT7 vector. The Y1H results showed that the MeABL5 protein successfully bound to the −800 to −400 bp region (P2) of the *MeCWINV3* promoter, and deletion of the ABRE binding site within P2 abolished their interaction in yeast (Figure 7B), suggesting that the MeABL5 protein recognizes the ABRE *cis*-element in the *MeCWINV3* promoter. The ABRE cis-element of CACGTG for MeABL5-binding proposed by the Y1H assay was further confirmed by EMSA assay. The wild-type sequence of CACGTG was bound by MeABL5, while the mutant-type sequence with CACGTG deletion could not be bound by MeABL5 (Figure 7C). Therefore, these results confirmed that MeABL5 binds to the ABRE *cis*-element of the *MeCWINV3* promoter.

### MeABL5 Positively Regulates the Activity of the *MeCWINV3* Promoter

**Figure 8:**
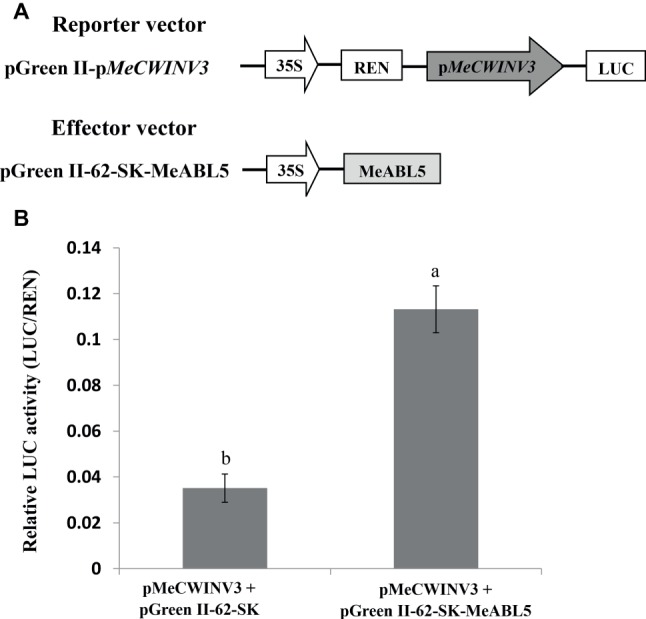
Regulation of the transcription activity of the *MeCWINV3* promoter by MeABL5 in a transient expression system. **(A)** Schematic diagrams of the vectors used in the transient expression analysis. **(B)** Transcriptional activity assay of the *MeCWINV3* promoter coinfiltrated with the effector vectors pGreen II 62-SK (control) or pGreen II 62-SK-MeABL5 into tobacco leaves. The *MeCWINV3* promoter was fused to the LUC reporter and the promoter activity was determined by a transient dual-LUC assay. The relative LUC activities were normalized to the reference Renilla (REN) luciferase. Error bars represent the SD of nine technical replicates. The significant difference was assessed by ANOVA; a and b represent a significant difference at *p* < 0.01.

A dual-luciferase assay system was employed to investigate the regulation of the *MeCWINV3* gene by MeABL5 *in planta*. The full-length cDNA of *MeABL5* was inserted into the effector vector (pGreen II 62-SK) and the *MeCWINV3* promoter was inserted into the reporter vector (pGreen II 0800-LUC) (Figure 8A); then, they were transiently coexpressed in tobacco leaves by *A. tumefaciens* GV3101-mediated transformation. Analysis of the relative luciferase activity (LUC/REN) revealed that the luciferase activity controlled by the *MeCWINV3* promoter was elevated by 3.2-fold compared to the activity of the control pGreen II 62-SK effector vector when MeABL5 was coexpressed in the infiltrated tobacco leaves (Figure 8B). These data indicate that transient overexpression of MeABL5 positively regulates the activity of the *MeCWINV3* promoter. Thereby, these results suggest that MeABL5 could be a positive regulator and active on the transcript level of the *MeCWINV3* gene in cassava.

### Expression Profiles Analysis of *MeABL5* and *MeCWINV3*

**Figure 9:**
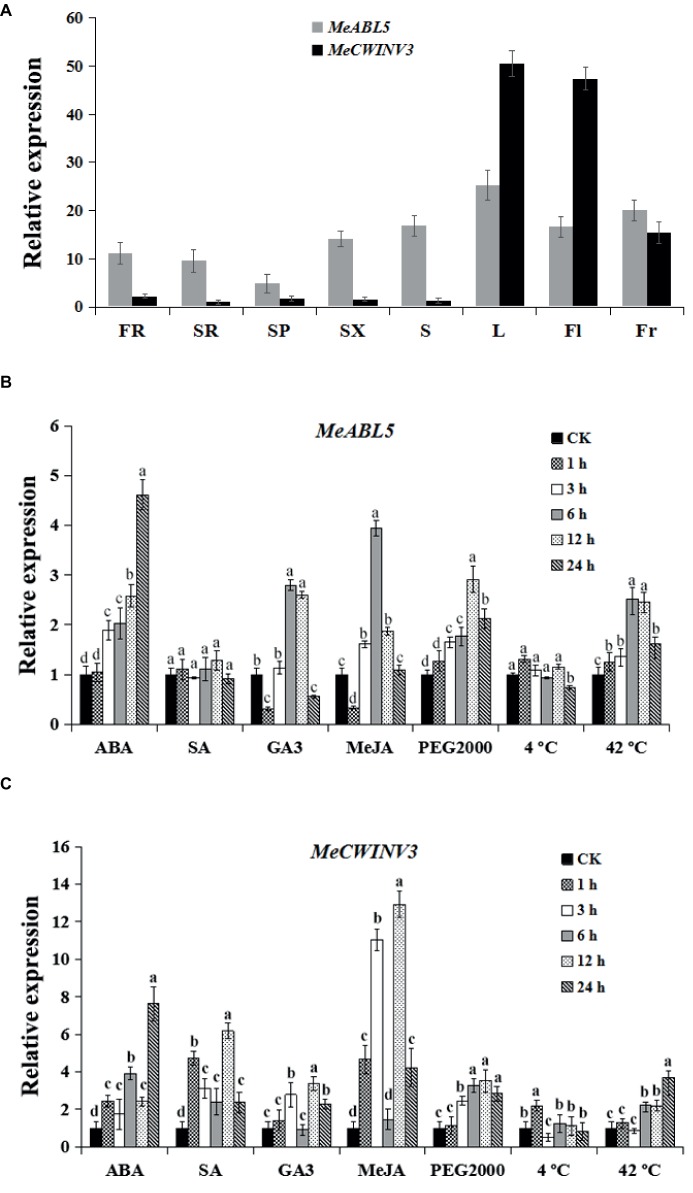
Expression pattern analysis of MeABL5 and MeCWINV3. **(A)** Expression analyses of MeABL5 and MeCWINV3 in cassava organs or tissues using qRT-PCR. FR, fibrous roots; SR, storage roots; SP, storage root phloems; SX, storage root xylems; S, stems; L, leaves (the seventh or eighth leaves from the top of the stem); Fl, flowers; Fr, fruits. The expression of MeCWINV3 in storage roots (SR) was used as a calibrator. **(B,C)** Expression patterns of MeABL5 **(B)** and MeCWINV3 **(C)** respond to ABA, SA, GA3, MeJA, PEG2000 (artificial drought condition), cold (4°C), and heat (42°C). Forty-five-day-old SC8 plantlets under different treatments were used for the analyses. The plantlets without any treatment were used as the control (CK). Each value represents the mean ± SE of three independent biological replicates. Letters a–d on the error bars indicate significant differences, as assessed by ANOVA (*p* < 0.05).

To determine the expression pattern of both *MeABL5* and *MeCWINV3* in various cassava organs and tissues, total RNAs were extracted separately from leaves, stems, flowers, fruits, fibrous roots, storage root phloems, and xylems for quantitative real-time PCR analysis. The data showed that *MeABL5* and *MeCWINV3* exhibited a similar expression pattern. Their expressions were detected in all the tested tissues, and both showed high levels of transcriptions in leaves, flowers, and fruits, with relatively less expression in the storage roots (Figure 9A). This result indicates that *MeABL5* and *MeCWINV3* function similarly throughout the whole developmental process of cassava.

To explore the possible involvement of *MeABL5* and *MeCWINV3* in response to abiotic stress in cassava, the expressions of *MeABL5* and *MeCWINV3* under exogenous hormones (ABA, SA, GA3, or MeJA) and abiotic stress conditions (PEG2000 artificial drought, 4°C low temperature or 42°C high temperature) were examined in 45-day-old tissue cultured plantlets of SC8 by qRT-PCR. The expressions of *MeABL5* were strongly induced by ABA and MeJA and reached the most dramatic induction levels at 24 and 6 h, respectively. *MeABL5* expression was also marginally stimulated by GA3. Similarly, the expression of *MeABL5* was induced by drought and reached its highest level after 12 h of PEG2000 treatment. Under 42°C heat conditions, the transcript level of *MeABL5* also showed rapid induction. By contrast, there were no obvious changes in expression of *MeABL5* under exogenous SA and 4°C low-temperature treatment (Figure 9B). The expressions of *MeCWINV3* were strongly induced by ABA and MeJA and moderately induced by SA, GA3, and 42°C heat conditions, but PEG2000 drought stress and 4°C low temperature had negligible effects on its expression (Figure 9C). In brief, a roughly similar expression pattern was shown for *MeABL5* and *MeCWINV3* under the abiotic stresses in cassava. Their expressions were both induced by ABA, GA3, MeJA, and 42°C heat, but non-responsive to 4°C low temperature. Nevertheless, they made the differences in response to exogenous SA and PEG2000 artificial drought stresses; the expression of *MeCWINV3* was up-regulated under SA treatment, while *MeABL5* was not sensitive to SA. Under drought conditions, the transcript level of *MeCWINV3* appeared to slightly decline, but the expression of *MeABL5* was moderately up-regulated (Figures 9B,C). These stress-induced expression profiles of *MeABL5* and *MeCWINV3* suggest that both of them are involved in abiotic stress response in cassava, but they might also exist as nuances of the function under certain abiotic stresses.

### MeABL5 Positively Regulates the Expression of *MeCWINV3* in Cassava

**Figure 10:**
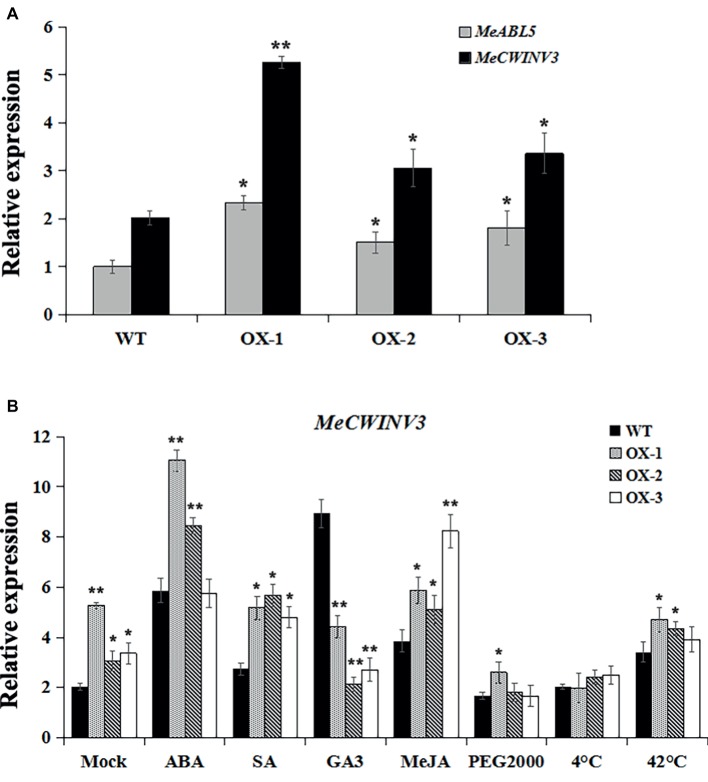
Regulation of the MeCWINV3 expression by MeABL5 in the overexpressing transgenic cassava FECs. **(A)** Expression analyses of MeABL5 and MeCWINV3 in the MeABL5-OX transgenic cassava FECs using qRT-PCR. Three independent transformation experiments were performed. The expression of MeABL5 in the untransformed cassava wild-type SC8 FECs (WT) was used as a calibrator. **(B)** Expression analyses of MeCWINV3 in the MeABL5-OX transgenic FECs under ABA, SA, GA3, MeJA, PEG2000, cold (4°C), or heat (42°C) treatment. The WT and MeABL5-OX transgenic FECs under different treatments for 12 h were used for the analyses. WT cassava SC8 FECs were used as control. The data are presented as the mean ± SE values (*n* = 3) from three independent experiments. Asterisks indicate statistically significant differences compared with WT. *p < 0.05; **p < 0.005.

The MeABL5 protein was able to bind and regulate the *MeCWINV3* promoter in both yeast and plants, and it was hypothesized that the expression of *MeCWINV3* was activated when MeABL5 transcript levels increased. It is difficult to obtain transgenics in cassava because of the lack of an efficient and robust transformation and regeneration system. To test whether MeABL5 can regulate the expression of *MeCWINV3* in cassava, cassava cultivar SC8 FECs were co-transformed with *MeABL5* overexpression vector CaMV35S::MeABL5. Three independent overexpression (OX) experiments were performed and incubated at 28°C at a 16/8-h photoperiod with carbenicillin and hygromycin selected for 7 days and further detected by qRT-PCR. The expression levels of *MeABL5* in the three *MeABL5*-OX FECs were 2.3-, 1.5-, and 1.8- fold of those in the wild-type control, while the expression of *MeCWINV3* gene showed a corresponding increase in the OX FECs (Figure 10A). The result supports the idea that MeABL5 positively regulates *MeCWINV3* expression in cassava.

To further evaluate the MeABL5 potential regulation effect of *MeCWINV3* under abiotic stresses, we examined the expression patterns of *MeCWINV3* in both *MeABL5*-OX and wild-type FECs treated with hormones (100 μM ABA, 2 mM SA, 100 μM GA3, or 100 μM MeJA) and abiotic stresses (20% PEG2000, 4°C or 42°C) for 12 h. The expressions of *MeCWINV3* under abiotic stresses in wild-type cassava FECs displayed similar patterns as in cassava plantlets. Its expression levels were induced by ABA, SA, GA3, MeJA, and 42°C heat, but were not stimulated by PEG2000 drought and 4°C cold stresses (Figure 10B). Interestingly, the *MeABL5-*up-regulated expressions of the *MeCWINV3* gene were obviously activated under ABA, SA, and MeJA treatments in *MeABL5*-OX FECs. However, *MeCWINV3* expression was not significantly induced by GA3 in *MeABL5*-OX FECs compared to wild type. Moreover, under the drought or cold stress conditions, the transcript levels of *MeCWINV3* were decreased to basal levels as in wild type. The results suggested that MeABL5 might act as a hormone (such as ABA, SA, or MeJA)-inducible activator to regulate the expression of *MeCWINV3*. These data also indicated that MeABL5 might play possible functions in mediating various abiotic stresses through different regulation pathways in cassava.

## Discussion

Transcription factors are proteins that can bind to promoters and work alone or with other proteins in a complex to regulate the transcription of downstream genes. The bZIP transcription factor family is one of the largest and most conserved families in plants. A total of 77 bZIP family members from cassava have been identified and clustered into 10 subfamilies (Hu et al., 2016b). In this study, a cassava bZIP transcription factor, MeABL5, was identified by an Y1H screen using the cassava *MeCWINV3* promoter as bait (Figure 1). MeABL5 is a cassava bZIP transcription factor that is not included in the 77 previously identified cassava bZIP family members and belongs to subfamily A (Figure 3). MeABL5 shows the closest homology to AtbZIP13 (AT5G44080) and AtbZIP40/GBF4 (AT1G03970) in Arabidopsis, and OsbZIP12/OsABF1 (Os01g64730) and OsbZIP40 (Os05g36160) in rice, which were previously classified as the subfamily A bZIPs (Hossain et al., 2010). The ABI5 or ABI5-like proteins in Arabidopsis and other species also have been reported to be classified as part of the subfamily A with their conserved sequence similarity (Choi et al., 2000; Uno et al., 2000). Subcellular localization and transactivation assays further revealed that MeABL5 is localized to the nucleus (Figure 4) and possesses transactivation activity (Figure 5). These data are consistent with what has been demonstrated for OsbZIP12/OsABF1 (Hossain et al., 2010) and other ABI5-like bZIP transcription factors such as the rice OsABI5 (Zou et al., 2008) and AtABI5 (Bensmihen et al., 2005). Similar to MeABL5, they all localize in the nucleus and function as transcription factors.

Numerous studies have confirmed the roles of bZIP transcription factors in the ABA response or in environmental adaptation through their regulation of the expression of stress-dependent genes and related physiological processes (Jakoby et al., 2002; Wei et al., 2012). The function of OsbZIP12/OsABF1 in rice has been intensively studied. OsABF1 is involved in abiotic stress responses and ABA signaling (Hossain et al., 2010); its expression is induced by abiotic stresses, ABA, sugar, and drought stress (Joo et al., 2014), and knockdown of both *OsABF1* and its closest homologous gene, *OsbZIP40*, results in a significantly earlier flowering phenotype in rice (Zhang et al., 2016). *BrABI5b*, an *AtABI5* ortholog, has been identified in *B. rapa* to be induced by drought and salt and plays a principal role in the adaptation to unfavorable conditions (Bai et al., 2016). *FaABI5* was shown to regulate fruit ripening and the response to salinity and UV-B radiation in strawberries (*Fragaria x ananassa* Duchesne) (Li et al., 2016a). Rice *ABI5-like 1* (*ABL1*) modulates ABA and auxin responses, and its expression could be induced by ABA, GA, indole-3-acetic acid, salinity, and drought but was not affected by cold stress (Yang et al., 2011). Similar to most bZIP subfamily A genes, cassava *MeABL5* was expressed in various cassava organs and tissues and could also be induced by ABA, GA3, MeJA, drought stress, and heat in our study (Figure 9B). This indicates a possible role for *MeABL5* throughout the developmental process and in response to abiotic stresses in cassava.

Plants are continuously exposed to a variety of stress factors in their natural environments, and different resistance mechanisms for abiotic and biotic stress are deployed to maintain their fitness (Roux et al., 2014; Mickelbart et al., 2015; Kissoudis et al., 2016). CWIs are essential enzymes that irreversibly hydrolyze sucrose into glucose and fructose to maintain hexose homeostasis in plants; hexose homeostasis plays a principal role in plant development and stress-related responses *via* sugar metabolism and other signaling pathways (Rolland et al., 2006; Ruan, 2014). There are six CWIs in cassava; the activity of MeCWINV1 and 3 was higher than that of other MeCWINVs in the cassava source and sink organs (Yao et al., 2014). *MeCWINV3* is highly expressed in leaves, especially mature leaves, in response to diurnal rhythm, and affects cassava productivity *via* regulating sugar allocation from source to sink (Yan et al., 2019). At present, in our current understanding, reports on the *MeCWINV3* concentrated on its function in the hydrolysis of sugar during phloem unloading to control plant development and sink strength, whereas rarely studies paid attention to its abiotic stress resistance in cassava.

Our present study has found that MeABL5 can specifically bind to the ABRE cis-element of the *MeCWINV3* promoter *in vitro* and *in vivo* and significantly up-regulates the activity of *MeCWINV3* promoter (Figures 6–8). *MeABL5* and *MeCWINV3* exhibited similar expression patterns in various organs or tissues and under abiotic stress in cassava (Figure 9). Their expressions were detected in all the tested tissues, and both showed high levels of transcriptions in leaves, flowers, and fruits, and relatively less expression in the storage roots. It indicates that *MeABL5* and *MeCWINV3* function similarly throughout the whole cassava developmental process and are involved in abiotic stress response in cassava. They might also exist as nuances of the function in some different stress conditions, such as SA and drought. Overexpression of *MeABL5* increased the activity of the *MeCWINV3* gene, and the up-regulated expressions of *MeCWINV3* were significantly activated under ABA-, SA-, and MeJA-induced conditions. The results strongly indicate that *MeABL5* is a positive regulator of *MeCWINV3* and might participate in the robust resistance of cassava in response to abiotic stress through the activation of CWI activities and the accumulation of hexose in cassava. This study also provides a foundation for further research on the ABA-mediated and stress-related signaling pathway in cassava.

## Conclusion

A bZIP transcription factor gene, designated as *MeABL5*, was isolated from cassava and belongs to bZIP subfamily A. MeABL5 is a nuclear-localized protein and possesses transactivation activity. MeABL5 specifically binds to the ABRE *cis*-element of the *MeCWINV3* promoter and significantly up-regulates the activity of the *MeCWINV3* promoter. *MeABL5* and *MeCWINV3* generally exhibited similar expression patterns in the tested organs/tissues and under the abiotic stress in cassava. Overexpression of *MeABL5* increased the *MeCWINV3* gene activity, and the up-regulated expression activities of *MeCWINV3* were significantly elevated under ABA-, SA-, and MeJA-induced conditions. Collectively, we hypothesize that *MeABL5* is a positive regulator of *MeCWINV3* and might contribute to the robust resistance of cassava to abiotic stresses.

## Author Contributions

JL, XC, and JG designed the research and wrote the manuscript. JL, XC, SW, YW, and YO performed the research. XC, YY, RL, SF, and XH modified the manuscript. All authors read and approved the final manuscript.

### Conflict of Interest Statement

The authors declare that the research was conducted in the absence of any commercial or financial relationships that could be construed as a potential conflict of interest.

## Footnotes

^1^http://www.UniProt.org/

^2^http://www.phytozome.net/cassava.php
