# Supplementary material for: MeABL5, an ABA Insensitive 5-Like Basic Leucine Zipper Transcription Factor, Positively Regulates MeCWINV3 in Cassava (Manihot esculenta Crantz)
Source: Front Plant Sci. 2019 Jun 28;10:772. doi: 10.3389/fpls.2019.00772 (PMC6609874; doi:10.3389/fpls.2019.00772)
Supplement: Supplementary file 1 [file Data_Sheet_1.PDF]

## *Supplementary Material*

**Supplementary Table S1. List of the *MeCWINV3* primers used in this study.**

| Primer Name | Sequence (5’ to 3’) | Function |
|---|---|---|
| pMeCWI3-A-F | CTGGTACCATCATTCTCACAGGAC | Bait vector construction |
| pMeCWI3-A-R | CAGTCGACAGCTCCTTTGATTGAAT | |
| pMeCWI3-P1-F | CTGGTACCCTGTTAGATAAAAT | Bait vector construction |
| pMeCWI3-P1-R | CAGTCGACAGCTCCTTTGATTGAAT | |
| pMeCWI3-P2-F | CTGGTACCTTTACTATGCTGTTC | Bait vector construction |
| pMeCWI3-P2-R | CAGTCGACCAGCTGATAGGCCA | |
| pMeCWI3-P3-F | CTGGTACCATCATTCTCACAGG | Bait vector construction |
| pMeCWI3-P3-R | CAGTCGACTTGAATATATATTA | |
| pMeCWI3-P2△ ABRE-F | TGACGTATCGGAAAGGGAAG | Bait vector construction |
| pMeCWI3-P2△ ABRE-R | CTTCCCTTTCCGATACGTCA | |
| pMeCWI3-F | ATCATTCTCACAGGACATC | EMSA |
| pMeCWI3-R | AGCTCCTTTGATTGAATAAG | |
| pMeCWI3-luc-F | CTGGTACCATCATTCTCACAGGAC | Dual-luciferase assay |
| pMeCWI3-luc-R | GTGGATCCAGCTCCTTTGATTG | |
| MeCWI3-qPCR-F | GCTTCGTGTGAATCCAGTCC | qRT-PCR analysis |
| MeCWI3-qPCR-R | TCTGCCTGTGATGCTGTGA | |

**Supplementary Table S2. List of the *MeABL5* primers used in this study.**

| Primer Name | Sequence (5' to 3') | Function |
|---|---|---|
| MeABL5-AD-F | TACATATGGCGTCGTCGAAG | Prey vector construction |
| MeABL5-AD-R | GCGGATCCTTAGCAGCCAAACAG | |
| MeABL5△ bZIP-AD-F | TACATATGGCGTCGTCGAAG | Prey vector construction |
| MeABL5△ bZIP-AD-R | GCGGATCCTGCTGGGTGGCCTT | |
| MeABL5-qPCR-F | GGGGATGACTTTGGAGGATT | qRT-PCR analysis |
| MeABL5-qPCR-R | CTCCTCTTCTGCCTCCCTTT | |
| MeABL5-GFP-F | TAGTCGACATGGCGTCGTCGAA | Subcellular localization |
| MeABL5-GFP-R | GCGGATCCAAGTTCTAAACAAAC | |
| MeABL5-BD-F | TACATATGGCGTCGTCGAAG | Transactivation activity |
| MeABL5-BD-R | GCGGATCCTTAGCAGCCAAACAG | |
| MeABL5-MBP-F | CAAGCTTGATGGCGTCGTCGA | Protein expression |
| MeABL5-MBP-R | CCTCGAGGTTAGCAGCCAAACAG | |
| MeABL5-SK-F | TAGAGCTCATGGCGTCGTCGA | Dual-luciferase assay |
| MeABL5-SK-R | GCGGATCCTTAGCAGCCAAACAG | |

1 atggcgtcgtcgaaggttctggcgacctcgtcaacgacgaatcctgatatgccacgtcaaccc

M A S S K V L A T S S T T N P D M P R Q P

64 tctttatgttcctccctctcaaccctgctcgccgatctccaaaaccaaaaccagttctcttct

S L C S S L S T L L A D L Q N Q N Q F S S

127 aattctcagagccctttgctctccgcgaccatggacgatctattgaaaaacatatactcttat

N S Q S P L L S A T M D D L L K N I Y S Y

190 cctactccaccgacaccagacgatccccacgcgcccccttctccggtggcgcgtcgatctca

P T P P T P D D P H A P P F S G G A S I S

253 cgcgatggtagcttccctttgcctaaggaagctgctagcaagtccgttgatgacgtttggaag

R D G S F P L P K E A A S K S V D D V W K

316 gaaattgtggccggcggggatcaccggcgtgatgaaaatggtggtggtggaggaatcgagggg

E I V A G G D H R R D E N G G G G G I E G

379 atgactttggaggattttctgactaaggccggtgcagtgagggaggaggatgttagaggggtt

M T L E D F L T K A G A V R E E D V R G V

442 ggaattccggttcaggtgggggctgctgtaggagcttatggtgtggatagcaatagcaaaata

G I P V Q V G A A V G A Y G V D S N S K I

505 actaatgagaataatagtaattatgatactggagaatttcaggggttgggaaatgggatgatg

T N E N N S N Y D T G E F Q G L G N G M M

568 gtggtggcagaaggagggaaagggaggcagaagaggagggctgtggaagaacctccaatggat

V V A E G G K G R Q K R R A V E E P P M D

631 aaggccacccagcagaagcagaggagaatgatcaagaatcgtgaatctgctgccaggtctagg

K A T Q Q K Q R R M I K N R E S A A R S R

694 gaacgtaaacaggttgtttttctttcttatttttctgatacttttattttcatgtttatgttt

E R K Q V V F L S Y F S D T F I F M F M F

721 ctgtttggctgctaa

L F G C *

**Supplementary Figure S1. The coding sequence and deduced amino acid sequence of *MeABL5*.** The bZIP domain are shown in shadow, and the asterisk represents the translation termination codon.

| | |
|---|---|
| -1160 | ATCATTCTCACAGGACATCAAATTTATATTAGACTACCTTATTTTAATTT |
| -1110 | CATTACCTAAATAATTAATATTAATAATTAAACTTTAGTGTTTGGAATAA |
| -1060 | TTAGCATTTTGGATTTTATCACTTATTTTCTTTATGTGAGAATGAGATGG |
| -1010 | ATACCTTTCTTGCTATAAATTCTAAATATCCCTTTTGACTTTGTTAATTT |
| -960 | ATTTTATAAAAAAAATTTAATTACAAATTATTATTATGTATATTTAAATT |
| -910 | ATACATATTAAAAATAAATAATTTTTAATAATATCTTTTAATAAAATTAA |
| -860 | ATATTTTTTAAATCAAAATTAAATCTTAATTAATAAAATAAAGTTTTAAT |
| -810 | ATATATTCAATTTACTATGCTGTTCCTTACGCTAAAATGAGCGGCATGCA |
| -760 | ATCGTTGACATGTGAGTATTTGACGTATCCACGTGGGAAAGGAAGAAAA |
| -710 | ACAACATTGAATAACCTAATTTTTGTCTAAATTCAATTTACATAAAAAAT |
| -660 | TCAAAATATAAATAAGAGTGTTTTATGTGATTTTAATTATATTAATAAAA |
| -610 | TCAAATTTCTTTTTTTTATTTTCATATTTAATTATTTTAATAACATTATAT |
| -560 | TTAGAAATATATAGAAATTGGGTTCACATGGTGAAGCTGTAAGACAACTA |
| -510 | GCATGGAAATAATTAAATAATTGTTTTTTTTTTTTCAAAATCCACCCAA |
| -460 | AAATCATTAATTGGTTGATTTACTGCCTTTGATAATTTGTCCCCTCTGGC |
| -410 | CTATCAGCTGCTGTTAGATAAAATAAAATATTCTATATATAAAATTTTTT |
| -360 | TAAAAATATATTATTATTCCAGAAATAAAGATATTTTTATATTACCTTAA |
| -310 | ACACATTGACTTTGACTTCAAAAACCAGTTTTCTTGGGTGCTGCTGTAAC |
| -260 | TTAGAAAGTGAAATTGAAATAAATAAATAGGATGGTTACCTGATTTTGAG |
| -210 | AACTTTTACTTCATTTTTGTTTTTCATATTTGAAAAAAATGAGAAAAACT |
| -160 | AGAAAAAATAATATTCCATGTAGACTAATCTTTTCATGCATCCACCAATT |
| -110 | TTCTCCATTAAGATAAAAATTTCTACTCTCTGCCACCAAACTCCTCTATA |
| -60 | TAAGTTCCTTGGGGAGTTGCAGACATGTCTCACTTCTCACCTTATTCAAT |
| -10 | CAAAGGAGCT atg |

**Supplementary Figure S2. The promoter sequence and the ABRE *cis*-element of *MeCWINV3*.** The ABRE *cis*-element is shown in shadow, and the box represents the translation start site ATG.
